# Antagonistic Roles of P2X7 and P2Y2 Receptors in Neurodegenerative Diseases

**DOI:** 10.3389/fphar.2021.659097

**Published:** 2021-04-12

**Authors:** Talita Glaser, Ágatha Oliveira-Giacomelli, Lyvia Lintzmaier Petiz, Deidiane Elisa Ribeiro, Roberta Andrejew, Henning Ulrich

**Affiliations:** Department of Biochemistry, Institute of Chemistry, University of São Paulo, São Paulo, Brazil

**Keywords:** motor disease, cognitive decline, neuroinflammation, ATP, UTP, neurogenesis, brain degeneration, brain repair, microglia

## Introduction

Purinergic signaling participates in physiological and pathophysiological processes in the developing and adult brain. Here, we discuss the state of the art of developments in P2 adenosine (ATP) and uridine (UTP) triphosphate activated purinergic receptor research and subsequent applications for the better understanding of the participation of purinergic signaling in neurodegeneration and neuroprotection.

In a seminal review in 2008, Burnstock (2008) already defined the most important features of purinergic P2 receptors in neurodegeneration, defining their roles in brain tissue damage by excessive extracellular ATP release, induction of neuroinflammation and interference in neural regeneration processes (scar formation, neuronal sprouting and adult neurogenesis). Professor Burnstock cited in this regard cerebral ischemia, Parkinson’s (PD), Alzheimer’s (AD) and Huntington’s (HD) diseases and concluded that hyper-expression and hyperactivities of some purinergic P2 receptors would be disease worsening, such as P2X7 receptors (P2X7R).

Neuroinflammation is strongly related to P2X7R activation, since danger associated molecular patterns (DAMPs), including extracellular ATP, are released (Ribeiro et al., 2020). Hence, ATP-induced activation of P2X7R prompts downstream release of proinflammatory mediators, such as interleukin 1β (IL-1β), through the assembling of the NLRP3 inflammasome (Bartlett et al., 2014). Thus, the NLRP inflammasome is followed by a downstream pro-apoptotic signaling cascade involved in neurodegenerative diseases (Heneka et al., 2010). Neuroinflammation involves the activation of microglia and astrocytes (Beamer et al., 2016), and the P2X7R can be found at highest density in the microglia (Weisman et al., 2012b). Its hyper-activation promotes the over-release of neurotransmitters from neuronal presynaptic terminals, contributing to apoptosis (Sperlágh and Illes, 2014). Further, we have shown that P2X7R are important inhibitors for neural phenotype determination using mouse pluripotent stem cells (Glaser et al., 2014; Glaser et al., 2020b), and promotion of glial differentiation (Yuahasi et al., 2012), aggravating tissue repair.

Inducing endogenous neurogenesis or implanting *in vitro* obtained neurons or their precursors are promising strategies for neurodegeneration prevention or neuronal recovery. Our laboratory has been contributing to the field of neurogenesis by detailed investigation of P2 receptor subtype expression and activity pattern along the course of neural differentiation (reviewed by Burnstock and Ulrich, 2011). Correspondingly, we clarified the neuroprotective features exerted by P2Y2 receptors (P2Y2R) during neural progenitor differentiation (Resende et al., 2008; Ulrich et al., 2012).

The P2Y2R is a G_q_-coupled receptor sensitive to activation by ATP and UTP/UDP, interacting with integrins and growth factor receptors (Weisman et al., 2012a) as well as inducing neuronal differentiation through activation of NGF/TrkA signaling, as shown for PC12 cells. Thus, this receptor can also promote proliferation of glioma C6 cells through the Ras–ERK pathway (Homolya et al., 1999; Arthur et al., 2005; Franke and Illes, 2006). Recently we demonstrated P2Y2R roles in favoring GABAergic phenotype determination (Glaser et al., 2020b). In addition, a novel concept in the neurodegeneration field characterizes the P2X7R as a trigger of pro-inflammatory responses, while the P2Y2R has neuroprotective properties. In this opinion article, we compare the role of both receptors in different neurodegenerative diseases we are working on.

## Balance of P2Y2R and P2X7R in Motor System-Related Diseases

In a previous review article, we discussed the intercorrelation between two basal ganglia disorders that compromise the motor system, HD and PD (Oliveira-Giacomelli et al., 2018; Glaser et al., 2020a). Reinforcing this idea, administration of the P2X7R antagonist Brilliant Blue G (BBG) could restore dyskinesia, a common symptom between both diseases (Fonteles et al., 2020).

### Huntington’s Disease

HD is a genetic degenerative and fatal disorder characterized by the loss of GABAergic neurons in the basal ganglia in the earlier stages, and extensive cortex degeneration later on, causing motor, cognitive and psychiatric dysfunctions. The mutation causing the disease consists of an expansion of repeated CAG triplets in the huntingtin gene (*HTT*), encoding for an expanded polyglutamine (polyQ) stretch.

Some previous works using mutant huntingtin expressing neurons or HD transgenic mouse models demonstrated roles for P2X7R in HD pathophysiology (Díaz-Hernández et al., 2009). *In vitro*, elevated levels of P2X7R and P2X7R-mediated calcium influx in soma and terminals of HD neurons increased susceptibility to apoptosis. *In vivo* administration of the P2X7R-antagonist BBG to a HD mouse model prevented neuronal apoptosis and attenuated body weight loss and motor-coordination deficits. In the last year, a study using postmortem striatum of HD patients corroborated the previous animal data. In both studies, the full-length form of the P2X7R protein and the naturally occurring trunked C-terminus region variant, showed upregulated expression levels. Taken together, P2X7R activity is prejudicial to brains of HD subjects.

In a recent work, we showed that neural precursor cells derived from embryonic stem cells regulate spontaneous calcium oscillations that control the translocation of phosphorylated CREB into the nucleus, activating the ASCL-1 pro-neuronal gene, thereby favoring neurogenesis towards the GABAergic phenotype (Glaser et al., 2020b). Consistently, our data from human neural precursor cells derived from induced pluripotent stem cells of HD subjects demonstrated impaired P2Y2R-mediated intracellular calcium mobilization and absent calcium spontaneous oscillations related to ASCL-1 activation (Glaser et al., 2020b).

### Parkinson’s Disease

PD is a motor disorder caused by the degeneration of the substantia nigra, thereby decreasing the amount of released dopamine in the basal ganglia. Due to intense neuroinflammation, mitochondrial dysfunction and neuronal degeneration of PD, the P2X7R has been extensively studied. PD patients exhibit the 1513A→C single nucleotide polymorphism in the P2X7R gene, which induces loss-of-function (Gu et al., 2001) and elevated risks of sporadic/late-onset PD development (Liu et al., 2013). In the striatal 6-hydroxydopamine (6-OHDA) lesion, increased binding of radioligands to the P2X7R was found in the striatum and substantia nigra of rodents (Crabbé et al., 2019). Corroborating these results, P2X7R gene expression was gradually increased within 5 weeks after forebrain bundle lesion by 6-OHDA in rats (Oliveira-Giacomelli et al., 2019).

Antagonism of P2X7R demonstrated promising results. 6-OHDA-injured rats treated with BBG restored dopaminergic fibers in the striatum and dopaminergic neurons in the substantia nigra (Ferrazoli et al., 2017; Oliveira-Giacomelli et al., 2019). Importantly, P2X7R blockade prevented motor impairment, mitochondrial dysfunction and dopamine deficit as well as decreased pro-apoptotic regulator expression and micro/astrogliosis induced by 6-OHDA (Marcellino et al., 2010; Carmo et al., 2014; Kumar et al., 2017; Oliveira-Giacomelli et al., 2019). BBG also alleviated dyskinesia, the aberrant balance between D1 and D2 receptors expression and micro/astrogliosis associated with L-DOPA treatment, the current gold standard treatment for PD (Fonteles et al., 2020).

*In vitro*, α-synuclein induced P2X7R activation in SH-SY5Y-derived dopaminergic neurons that modulated mitochondrial dysfunction, ATP release, recruitment of pannexin-1 and decreased ATP degradation, with consequent cell death (Wilkaniec et al., 2017, Wilkaniec et al., 2020).

### Preventing and Recovering Basal Ganglia Lesions

*As we highlighted here*, strong evidence points at the P2X7R as a neurodegeneration inducer for both PD and HD through neuroinflammation. Pharmacological tools, such as blockade of the P2X7R over activation by BBG, may prevent the further damage. We believe that endogenous neurogenesis inducers, such as the P2Y2R, may add therapeutic strength by promoting the delivery of newly born neurons from the subventricular zone to the site of degeneration and recovering the lesioned striatum and movement control.

## Balance of P2Y2R and P2X7R in Cognitive-Related Diseases

Both AD and epileptic seizures impair cognition and steadily damage hippocampal circuitry, leading to progressive memory loss. Neuronal hyperexcitability induced by seizures amplifies the synaptic release of the main component of senile plaques found in the brain of AD patients, such as beta amyloid peptide (Aβ), enhancing cell death and cognitive decline. Nowadays, strong evidence indicates epilepsy as a comorbidity of AD, which is corroborated by P2X7R and P2Y2R functions in these brain disorders (Noebels, 2011).

### Alzheimer’s Disease

P2X7R expression is upregulated in AD patients and animal models (Parvathenani et al., 2003; McLarnon et al., 2006; Ryu and McLarnon, 2008). Receptor expression augments in microglia surrounding Aβ plaques, occurring in parallel with AD progression (Lee et al., 2011). Aβ aggregation triggers neuroinflammation in AD, as patients may have Aβ deposits as early as 10 years prior to first AD symptoms (Vermunt et al., 2019). Upon ATP binding, the P2X7R activates microglia, leading to a proinflammatory state that can promote amyloid-precursor protein (APP) release and oxidative stress in AD pathology, leading to synaptic dysfunction/loss and cell death.

In SH-SY5Y neuroblastoma cells, BzATP stimulated the release of APP, and the use of antagonists or knockdown with siRNA confirmed P2X7R dependence of APP release (Delarasse et al., 2011). AD animal models showed that the P2X7R function is as necessary for Aβ deposition. The treatment with P2X7R antagonists decreased the size and number of 8 months old J20 mice hippocampal amyloid plaques (Diaz-Hernandez et al., 2012), while 10 months old P2X7R knock-out APP/PS1 mice displayed less Aβ lesions, improved cognitive deficits and synaptic plasticity (Martin et al., 2019). Aβ plaques promoted the release of IL-1β, an event depending on P2X7R activation (Sanz et al., 2009). This occurred at least in part through Aβ-induced generation of pore-like structures, allowing ATP leakage into extracellular environments and binding to P2X7R, enhancing excitatory synaptic activity (Sáez-Orellana et al., 2016). The inflammatory process resulting from hyperexcitability is one of the key factors in AD (Busche and Konnerth, 2015). Lastly, high levels of reactive oxygen species (ROS) are commonly detected in *postmortem* brains of AD patients (Tönnies and Trushina, 2017). P2X7R activation is associated with Aβ-induced microglial H_2_O_2_ release through NADPH oxidase activation (Soo et al., 2007). P2X7R-positive microglial cells located around Aβ plaques expressed the catalytic NADPH subunit, and P2X7R upregulation combined to ROS release was associated to Aβ deposition increase and synaptotoxicity in AD (Lee et al., 2011).

Adversely, *in vitro* and *in vivo* studies corroborate neuroprotective roles of P2Y2R activation in AD. *Postmortem* studies showed that P2Y2R immunoreactivity is preserved in the occipital cortex (minimally affected region) while it is reduced in the parietal cortex (highly affected region) of AD patients. Interestingly, decreased expression of P2Y2R in the parietal cortex is correlated with AD neuropathologic scores and markers of synapse loss (Lai et al., 2008).

In human 1321N1 astrocytoma cells, P2Y2R stimulation enhanced non-amyloidogenic processing of APP. Corroborating these results, P2Y2R activation in rat primary cortical neurons treated with IL-1β enhanced the release of α-amyloid protein (Kong et al., 2009). In addition, treatment with P2Y2R agonists (ATP and UTP) enhanced the uptake and degradation of Aβ, while Aβ application increased P2Y2R gene expression in mouse primary microglial cells (Kim et al., 2012). The role of P2Y2R has also been investigated in transgenic mice bearing human APP with Swedish and Indiana mutations, an animal model of AD. In these animals, haploinsufficiency of P2Y2R augmented plaque formation and enhanced Aβ levels in the cerebral cortex and hippocampus as well as led to neurological deficits within 10 weeks (Ajit et al., 2014). Moreover, P2Y2R deletion induced premature death in these transgenic mice (Ajit et al., 2014).

### Epilepsy

Epilepsy can impact cognitive function, since the seizures cause excitotoxicity and cell death (Helmstaedter, 2013). Similarly to AD, P2X7R protein levels are upregulated in regions damaged by seizures and in the hippocampus of animal models. As previously summarized (Engel et al., 2012; Engel et al., 2016), the lack of the P2X7R promotes susceptibility to status epilepticus, while P2X7R antagonists are potent anticonvulsants (Engel et al., 2012; Engel et al., 2016; Beamer et al., 2017; Zeng et al., 2017; Burnstock and Knight, 2018; Song et al., 2019; Doǧan et al., 2020; Hong et al., 2020; Morgan et al., 2020). P2Y2R knockout animals present higher glutamate release in the hippocampus (Alhowail et al., 2020), and uridine triphosphate administration had sleep-promoting and anti-epileptic actions, improved memory function and affected neuronal plasticity (Dobolyi et al., 2011; Alves et al., 2017).

### Preventing and Restoring Cognition

Both AD and Epilepsy harm cognition capabilities, mainly through microglial activity, thus damaging the hippocampus. P2X7R sensitization promotes the accumulation of plaques, while P2Y2R activity enhances their uptake and degradation. In this case, we have two major players, the good and the evil, one stimulated by ATP and the other by UTP, like a Yin Yang effect.

## Discussion and Perspectives

**FIGURE 1:**
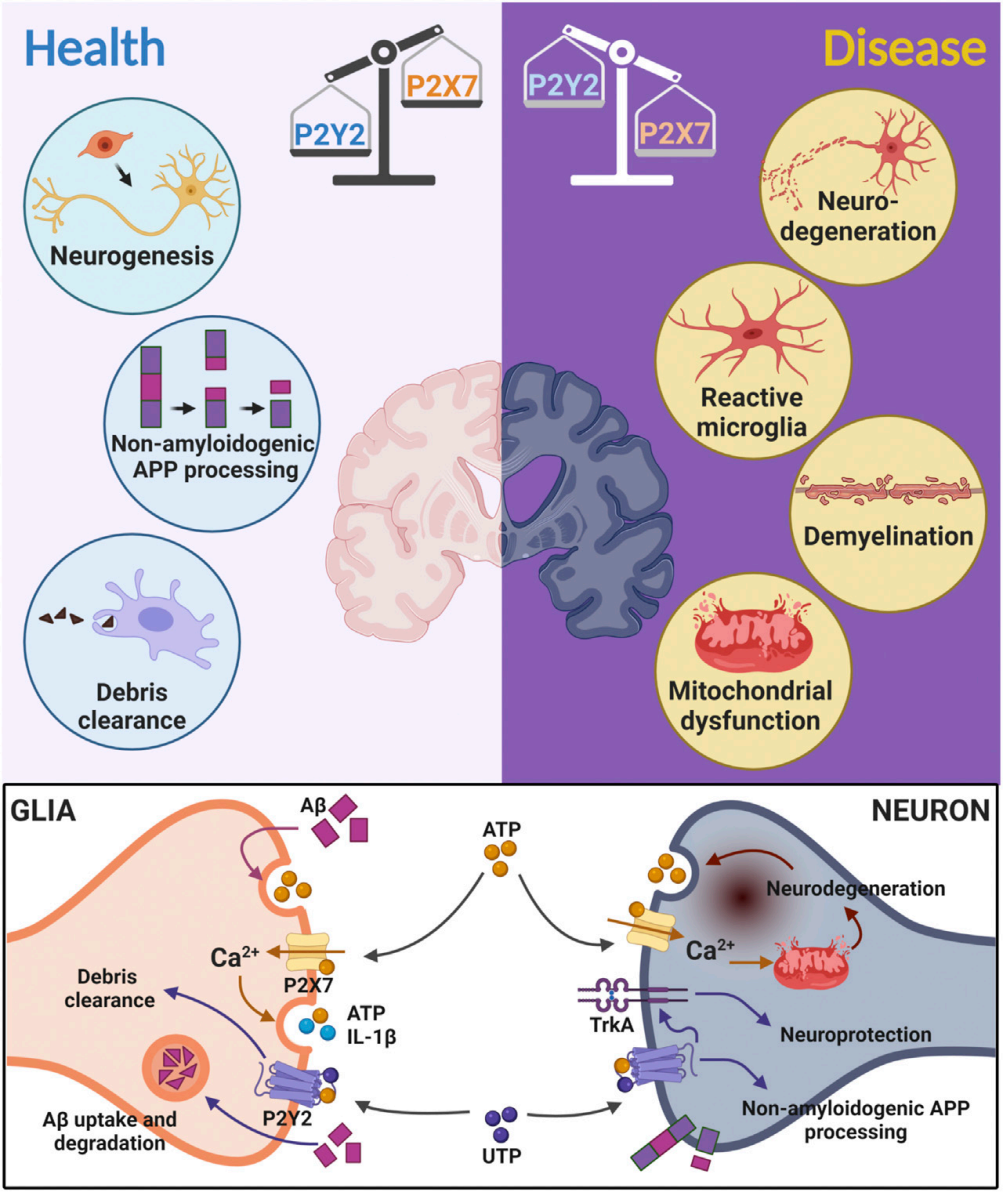
The yin and yang effects of P2Y2R and P2X7R activities. A thin balance between P2Y2R and P2X7R activities is required to maintain a healthy communication between neural cells. Increased P2Y2R activation (Health—**left**) induces neuroprotective effects, like neurogenesis, clearance of debris from apoptotic cells and amyloid precursor protein (APP) non-amyloidogenic processing in Alzheimer’s Disease. P2X7R expression and activity is augmented in the diseased brain (Disease—**right**), inducing neurodegeneration, microglia activation, demyelination, and mitochondrial dysfunction. At the cellular level, ATP is intensely released during neurodegeneration. Released ATP by activating P2X7R increases intracellular Ca^2+^ levels and can induce detrimental effects such as mitochondrial dysfunction and neurodegeneration. In glial cells, P2X7R activation induces the release of more ATP and interleukins, such as IL-1β. ATP and UTP can bind to P2Y2R inducing neuroprotective effects, including: debris clearance by glial phagocytosis; increase in the sensibility of TrkA receptors and stimulation of the neural growth factor pathway; amyloid-β (Aβ) uptake and degradation as well as non-amyloidogenic APP processing (in Alzheimer’s Disease). Increased ATP release can also be triggered by Aβ exposure. Created with BioRender.com.

Pharmacological intervention of purinergic signaling provides promising therapeutic avenues. We focused in this Opinion article on beneficial and harmful actions of purinergic receptors, affecting neuroinflammation and neuronal repair (Figure 1). In this scenario, two main players have been identified: 1) The P2X7R, known to counteract neuronal differentiation during development, which supposedly has similar effects on adult neurogenesis and also limits the available neural stem cell pool (Oliveira et al., 2016). This receptor is also directly connected to ATP induced inflammasome activation, mediating sterile inflammation of the brain; 2) The P2Y2R, shown to promote neuronal differentiation and neuronal phenotype determination of stem cells, has been associated with neuroprotective features in various brain disorders, including AD, HD as well as in epilepsy. The P2Y2R is highly expressed in axonal projections in the striatum and substantia nigra (Amadio et al., 2007) as well as in microglia, mediating the uptake of toxic peptides such as Aβ (Kim et al., 2012). In this sense, we hypothesize that the P2Y2R participates in the phagocytic process of α-synuclein in PD. However, the P2Y2R is unexplored in the PD pathogenesis, evidencing a gap in the literature and a promising target for future research. IL-1β induces P2Y2 receptor expression (Peterson et al., 2013); thereby, we propose that P2Y2R activity acts as an effort to restore brain homeostasis.

Several BBB-permeant small molecule P2X7R antagonists were developed (for a review, see Andrejew et al., 2020), and some of them are undergoing clinical trials for the treatment of neurodegeneration: CE22,535, already tested in phase 2 and 3 clinical trials; and JNJ541754467 tested in a phase 1 clinical trial (for a detailed review, see Calzaferri et al., 2020). Therapeutic P2Y2R agonists include diquafosol (Lau et al., 2014) and Denufosol tetrasodium (Deterding et al., 2007). However, BBB-permeant P2Y2R activators need yet to be developed. P2X7R antagonists and P2Y2R agonists could be administered alone or in combination with conventional drug therapy. An interesting pharmacological approach would be based in the combination of P2X7R antagonist and P2Y2R agonist.

## Abbreviations

6-OHDA, 6-hydroxydopamine; Aβ, amyloid-β; AD, Alzheimer’s disease; APP, amyloid-precursor protein; ATP, adenosine triphosphate; BBB, blood-brain-barrier; BBG, Brilliant Blue G; DAMPs, danger associated molecular patterns; HD, Huntington’s disease; IL-1β, interleukin 1β; P2X7R, purinergic P2X7 receptor(s); P2Y2R, purinergic P2Y2 receptor(s); PD, Parkinson’s disease; ROS, reactive oxygen species; UTP/UDP, uridine tri-di-phosphate
